# Efficacy of novel allogeneic cancer cells vaccine to treat colorectal cancer

**DOI:** 10.3389/fonc.2024.1427428

**Published:** 2024-07-24

**Authors:** George Alzeeb, Corinne Tortorelli, Jaqueline Taleb, Fanny De Luca, Benoit Berge, Chloé Bardet, Emeric Limagne, Marion Brun, Lionel Chalus, Benoit Pinteur, Paul Bravetti, Céline Gongora, Lionel Apetoh, Francois Ghiringhelli

**Affiliations:** ^1^ Brenus-Pharma, Lyon, France; ^2^ Imthernat, Université Claude Bernard Lyon 1, Therapies and Immune REsponse in Cancers (TIRECs), Lyon, France; ^3^ Bio Elpida, Lyon, France; ^4^ Euraxi, Joué-lès-Tours, France; ^5^ Anaquant, Lyon, France; ^6^ Transfer Platform for Cancer Biology, Centre Georges François Leclerc, Dijon, France; ^7^ Institut de Recherche en Cancérologie de Montpellier, INSERM U1194, Université de Montpellier, Montpellier, France; ^8^ Brown Center for Immunotherapy, Indiana University Melvin and Bren Simon Comprehensive Cancer Center, Indiana University School of Medicine, Indianapolis, IN, United States

**Keywords:** cancer vaccine, stimulated tumor cells, colorectal cancer, antigens, haptenation, immune response, proteomics, immunotherapy

## Abstract

Colorectal cancer (CRC) remains a significant global health burden, emphasizing the need for innovative treatment strategies. 95% of the CRC population are microsatellite stable (MSS), insensitive to classical immunotherapies such as anti-PD-1; on the other hand, responders can become resistant and relapse. Recently, the use of cancer vaccines enhanced the immune response against tumor cells. In this context, we developed a therapeutic vaccine based on Stimulated Tumor Cells (STC) platform technology. This vaccine is composed of selected tumor cell lines stressed and haptenated *in vitro* to generate a factory of immunogenic cancer-related antigens validated by a proteomic cross analysis with patient’s biopsies. This technology allows a multi-specific education of the immune system to target tumor cells harboring resistant clones. Here, we report safety and antitumor efficacy of the murine version of the STC vaccine on CT26 BALB/c CRC syngeneic murine models. We showed that one cell line (1CL)-based STC vaccine suppressed tumor growth and extended survival. In addition, three cell lines (3CL)-based STC vaccine significantly improves these parameters by presenting additional tumor-related antigens inducing a multi-specific anti-tumor immune response. Furthermore, proteomic analyses validated that the 3CL-based STC vaccine represents a wider quality range of tumor-related proteins than the 1CL-based STC vaccine covering key categories of tumor antigens related to tumor plasticity and treatment resistance. We also evaluated the efficacy of STC vaccine in an MC38 anti-PD-1 resistant syngeneic murine model. Vaccination with the 3CL-based STC vaccine significantly improved survival and showed a confirmed complete response with an antitumor activity carried by the increase of CD8+ lymphocyte T cells and M1 macrophage infiltration. These results demonstrate the potential of this technology to produce human vaccines for the treatment of patients with CRC.

## Introduction

Colorectal cancer (CRC) is the third most common cancer (10% of new cases in 2020) and the second leading cause of cancer-related death (9.4%) worldwide (1). Metastatic colorectal cancer (mCRC) is diagnosed after the recurrence of 35% of CRC or at initial diagnosis. mCRC can be divided into two distinct categories: the first consists of tumors with proficient mismatch repair (pMMR) characterized by microsatellite stability (MSS) and represents 95% of mCRC cases (2). The second category comprises those with deficient mismatch repair (dMMR) with high level of microsatellite instability (MSI-H). Standard of care chemotherapy doublet are effective but with a toxicity not allowing long-term administration with a risk of recurrence or progression due to the well-known treatment resistances, tumor plasticity, and toxicity (3, 4). The challenge of current research is to counter resistance mechanisms and tumor plasticity to avoid residual disease progression and relapse mechanisms. Recent tremendous progress in immunotherapy, which involves bypassing the evasion strategies of malignant cells and boosting the immune system against them, opens multiple ways to address therapeutic challenges in mCRC (5). dMMR/MSI-H CRC have been determined to exhibit a higher tumor mutation burden than pMMR/MSS CRC and more tumor infiltrating lymphocytes (TILs) with activated CD8+ cytotoxic T-lymphocyte (CTL) and T helper type 1 (Th1) cells characterized by IFN-γ production: making these tumors sensitive to treatment with immune checkpoint inhibitors (ICIs), like anti-PD-1 (Programmed cell death protein 1, CD279) and anti-PD-L1 (Programmed Death Ligand 1, CD274) inhibitors (6–9). These therapies enhance the activity of immune cells against tumors. However, 95% of mCRC patients (pMMR/MSS) will not respond to these immunotherapies (10) and those who are initially sensitive to ICI will eventually relapse [until 29% (11)]. Different therapeutics approaches are in development phases like CAR-T cell therapy, mRNA cancer vaccine or proteins cancer vaccine, but are still limited concerning the panel of antigen presented to the immune system and have manufacturing challenges that may be a barrier for the patient’s access (12).

The whole-cell vaccines strategy has been explored as a potential immunotherapeutic option. The first trial of the autologous whole cell-based vaccine called ONCOVAX showed interesting results in patients with stage II colon cancer (13). Autologous vaccines are known to be time-consuming and difficult to produce, and their efficacy has shown varying results. To overcome these limitations, we aim to develop a new generation of therapeutic cancer vaccines based on whole allogeneic cells. These vaccines can be used for patients even when biopsies are not possible. The production of this family of vaccines will be achieved by the Stimulated Tumor Cells (STC) platform, which aims to simplify vaccine manufacturing. The STC vaccine is composed of characterized CRC cell lines that are selected to represent tumor patients. The cells are stimulated *in vitro* to replicate the stress of patient tumor cells, inducing the overexpression of tumor-related proteins such as resistance proteins, stress proteins, or tumor plasticity proteins. The second objective of the stimulation techniques is to increase the immunogenicity rates of the vaccine, such as the overexpression of damage-associated molecular patterns (DAMPs), including heat shock protein (HSP)-70 overexpression in inactivated tumor cells resulting from thermal stress. This process mediates phagocytosis and subsequent cross-presentation of foreign antigens to the immune system by interacting with their receptor CD91 (14). HSP alone was used as an anticancer vaccine (15–18). A further *in vitro* step of haptenation is added in the process of STC vaccine production to conjugate a chemical sequence to proteins, including antigens, with a strong covalent liaison (19). The hapten-conjugate aims to induce immunological potential of stimulated cells (20). The strong immunogenicity of haptens *in vivo* results from their capacity to directly activate or mature DC, or indirectly by stimulating the release of proinflammatory signals by epithelial cells (21). Research on skin sensitization in mice has demonstrated that TNF-α (produced by keratinocytes in response to haptens) and IL-1β are rapidly up-regulated after haptens are applied, working together to support the supply of DC (22). Hapten-induced tumor regression has been studied since the mid-1900s, Hamaoka, et al. describes a haptenation concept of patients’ tumor and injected back into sensitized animals or patients (23). Berd et al. employed ex vivo haptenation in several clinical trials, both as a primary treatment for metastatic melanoma and as an adjuvant therapy following surgical resection of nodal metastases in patients with stages III and IV metastatic melanoma. In 2004, Berd et al. extended the 1997 study to 214 patients with 5-year overall survival of 44% (24, 25). Haptenation has been demonstrated as an efficient strategy for increasing the immunological function of vaccines. These stimulated and haptenated tumor cells, composing the STC vaccine, are inactivated to block proliferation capability (dead cell with maintained membrane) and ready to use.

Herein we report 3 proof-of-concept studies in syngeneic murine models of CRC using a mouse surrogate vaccine generated using the STC platform. Different version of the STC vaccines have been produced to improve production strategies and to confirm vaccine efficacity and non-toxicity. We report results from the CT26 colon cancer model, aiming to: a) evaluate the efficacy of a one cell line (1CL)-based STC vaccine and to b) investigate the potential increase of antitumoral effect of a broader panel of cancer-related proteins expressed by 3 cell lines (3CL)-based STC vaccine. We established a proteomic approach to identify the increased panel of cancer-related proteins using 3CL instead of one. We evaluated efficacy and safety of the 3CL-based STC vaccine in MC38 with *in vivo* resistance to anti-PD1 model (MC38-PD1-R) (26). Vaccination using the 3CL-based STC vaccine led to a significant enhancement in survival rates, demonstrating a confirmed complete response characterized by heightened antitumor activity through increased infiltration of CD8+ T lymphocytes and M1 macrophages.

## Materials and methods

### Mice and cell lines

Female BALB/c mice (6- to 8-weeks-old) provided by the JANVIER laboratory (Saint Berthevin, France) were acclimatized for 7 days under standard conditions. Experiments on animals were conducted in an agreed-upon facility (IMTHERNAT, Lyon, France, agreement #A69388) after protocol approval by the Ethics Committee for Animals (UCBL1, Lyon, France, approval #DR2015-29). The animals were anesthetized for all injections and measurements.

Female C57BL/6 mice (4- to 5-weeks-old) were provided by the Charles River Laboratory (#000664). All mice were raised in a specific pathogen-free environment with free access to standard food and water. Experiments on animals were conducted in an agreed-upon facility (Antineo, Lyon, France) after protocol approval by the Animal Ethics Committee CECCAP of Lyon. No experiment lunched on mice aged less than 6 weeks.

The murine cancer cell lines: CT26 (ATCC (American Type Culture Collection) cell line number CRL-2639, derived from BALB/c mouse colon tumor), CMT-93 (cell line number 89111413, derived from rectal carcinoma from ECACC for European Collection of Authenticated Cell Cultures), LTPA (ATCC CRL-2389, from pancreatic adenocarcinoma), and murine colon cancer MC38 cells (Kerafast, USA).

### Preparation of grafts

#### CT26 BALB/c colorectal syngeneic murine model

CT26 cells were grown in RPMI medium supplemented with 10% fetal calf serum at 37°C under a humidified atmosphere with 5% CO_2_, trypsinized, washed, and resuspended in phosphate-buffered saline (PBS) (Lonza, France) with 5% dimethyl sulfoxide (DMSO) (from Sigma, France) at 5.10^5^ cells/mL. The obtained CT26.WT graft doses were frozen at -80°C. On the day of the graft (D0), all mice received a 100 µL subcutaneous (SC) injection of the graft dose at the left flank (5.10^4^ CT26.WT cells/mouse). Graft preparation and tumor growth (TG) in mice were similar in Studies A and B.

#### MC38 anti-PD1 resistant C57BL/6 colon carcinoma syngeneic murine model

The model was established as described before (26), briefly, one million MC38 cells of exponentially growing cultures, in DMEM medium with 10% fetal bovine serum, 100 U/mL penicillin and streptomycin, were diluted in 0.2 mL of PBS (Gibco, 140040-091) and injected subcutaneously into the left flank of C57BL6 mice. The tumor volume was measured twice a week (length x width) with a caliper. The tumor volume was determined using the formula: 4/3 x π x r^3^. When the tumor volume reached 150mm^3^, mice were randomized, and a treatment of anti-PD-1, to generate resistant tumor, was administered to groups of 5 to 6 mice and monitored for tumor growth and flow cytometry analyses. To establish the resistant models, tumors obtained from mice with initial responses to anti-PD-1 (BioXCell, RMP1-14, BE014, RRID: AB_10949053, 12.5 mg/kg/week) were serially reimplanted subcutaneously into new groups of naïve mice (in the right flank) and treated once a week by anti-PD-1 to maintain selection pressure. At each passage, three naïve mice were implanted with tumor fragments and treatment with anti-PD-1 was initiated once the tumor reached 150mm^3^. The most aggressive tumor was selected for reimplantation. At least five passages were necessary to induce acquired resistance.

### Vaccines production

**Figure 1:**
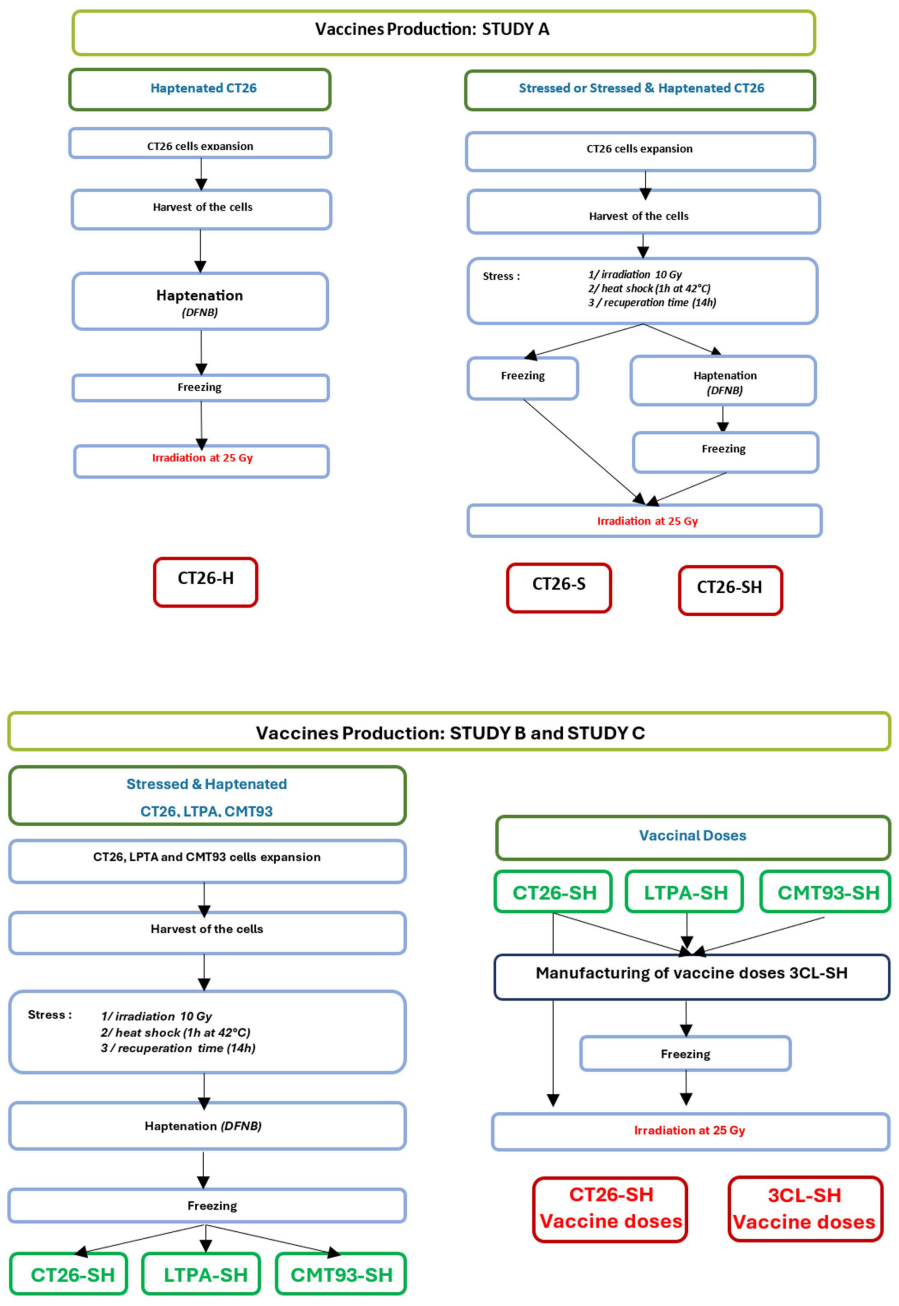
Manufacturing flowchart. Representation showing vaccines production steps for study A (one cell line-based vaccine: CT26) and study B & C (three cell lines-based vaccine: CT26, CMT-93 and LTPA). -S, stressed cells; -H, haptenated cells; -SH, stressed and haptenated cells.

Different vaccines were produced for these studies (**Figure 1**):

#### Study A: 1 cell line-STC based vaccine

CT26 cells were used to produce vaccine doses. First, cells were subjected to physical stimulation with low-dose irradiation (10 Gy) and mild thermal stress: 1h at 42°C then 14h at 37°C. Second, thermally stressed CT26 cells (CT26-S) were haptenated (H) using dinitrofluorobenzene (DFNB) (Sigma, France) solution at 12.7 µg/mL for 30 min, [adapted from (27)], to obtain haptenated and thermally stressed CT26 cells (CT26-SH). In addition, we generated haptenated CT26 cells (CT26-H) using the same protocol. CT26-S, CT26-H, and CT26-SH vaccine doses were frozen at -80°C in Earle’s Balanced Salt Solution (EBSS) (Thermo Fisher Scientific, Gibco) containing 80 g/L sucrose (VWR, France) before irradiation (25 Gy) at 10.10^6^ cells/mL.

#### Study B & C: 3 cell lines-STC based vaccine

Vaccines were produced using an equal mixture of three murine cell lines (3CL): CT26, CMT-93 and LTPA, which were stressed (S) and haptenated (H) following the same procedures for each cell line as described above for study A, to obtain stimulated and haptenated 3 tumor cell lines vaccine (3CL-SH). Vaccinal doses (10.10^6^ cells/mL) were frozen at -80°C in Earle’s Balanced Salt Solution (EBSS) containing 80 g/L sucrose before irradiation at 25 Gy.

Thermal stress was controlled by flow cytometry using fluorescence-activated cell-sorting to control the overexpression of HSP70 using a cmHSP70.1 monoclonal antibody (Klinikum, TU München) (**Supplementary Table 1**).

### Studies design

#### Study A

**Figure 2:**
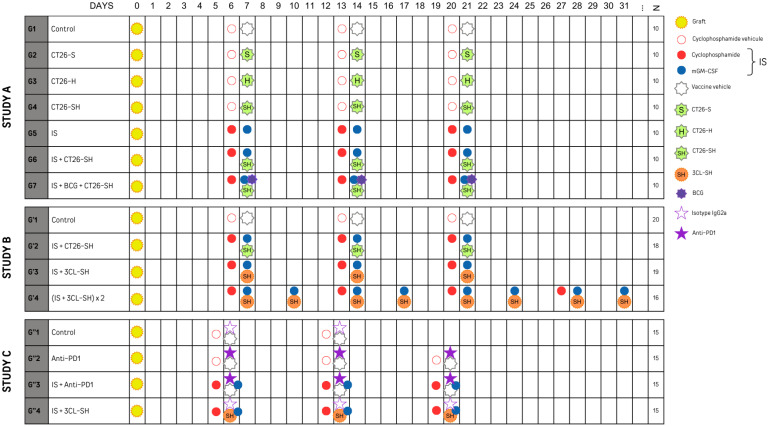
Experimental plan of studies A, B and C sets with N: number of mice per group. mGM-CSF: mouse granulocyte macrophage colony stimulating factor. IS, immunostimulant; 15 mg/kg CP and 5 µg mGM-CSF; 3CL, three cell lines; -S, stressed cells; -H, haptenated cells; -SH, stressed and haptenated cells.

Mice were allocated to seven groups (G1 to G7, n=10 per group) and treated accordingly to the experimental plan shown in **Figure 2**. The vaccine doses were thawed, washed, and resuspended in PBS. Vaccine subcutaneous (SC) injections were administered on D7, D14 and D21 (10^6^ cells/mouse/injection) in the right flank, whereas tumor cells were grafted subcutaneously into the left flank. Concerning the immune stimulants (IS), cyclophosphamide (Sigma, France) (CP, 15 mg/kg) was administered by intraperitoneal (IP) injection one day before vaccination, and mouse granulocyte-macrophage colony-stimulating factor (PeproTech, Neuilly-sur-Seine, France) (mGM-CSF, 5 µg) was administered by SC injections mixing with the vaccine. Group 7 received Bacillus Calmette-Guerin (Medac, Lyon, France) (BCG, 2.10^6^ CFU) in addition to the vaccine and IS.

#### Study B

Four groups G’1 (20 mice), G’2 (18 mice), G’3 (19 mice) and G’4 (16 mice)) were treated according to the experimental plan presented in **Figure 2**. G’1 and G’2 groups respectively restored the treatment plan of G1 and G6 groups from study A. For G’2 and G’3, vaccine injections were made similar to the first experiment at D7, D14, and D21. In contrast, for G’4 it was made 2 times per week for 4 weeks at D7, D10, D14, D17, D21, D24, D28, and D31. Similar to G6, G’2 mice received 10^6^ CT26-SH cells/mouse/injection; mice in G’3, and G’4 received 10^6^ 3CL-SH cells/mouse/injection, including equal amounts of each cell line (0.33x10^6^ CT26-SH + 0.33x10^6^ CMT-93-SH + 0.33x10^6^ LTPA-SH cells/mouse/injection). All groups except control group received IS. As in Study A, administration and transplantation were performed according to the same scheme. Mice were sacrificed when the tumors reached a maximum volume of 1000 mm^3^.

#### Study C

To evaluate the therapeutic efficacy in a context of acquired resistances to anti-PD-1, MC38 anti-PD1-R tumor fragments were implanted subcutaneously into the left flank of immunocompetent C57Bl/6 mice, mice were randomized into four groups of 15 mice and treated according to the experimental plan presented in **Figure 2**. Control group (G”1) was treated with the isotype IgG2a monoclonal antibody (mAb) (clone 2A3), mice in G”2 were treated with the mAb anti-PD-1 (anti-murine PD-1 clone CD279 RPMI-14). G”3 and G”4 received immune stimulants: CP (CFL Biotech) and mGM-CSF (PeproTech), in addition to the anti-PD-1 mAb or the 3-Cl-SH vaccine respectively. Vaccinal doses were thawed, washed, and resuspended in PBS. Vaccine injections were made on D6, D13 and D20 (10^6^ cells/mouse) at the right flank. The immune stimulation with SC injections of CP at 15 mg/kg and 5 µg mGM-CSF. The treatments occurred once per week for a total of three weeks. The animals were monitored throughout the study for weight and behavior. Injection sites were monitored for inflammatory reaction, and the tumor size was measured with a caliper three times per week. Mice were sacrificed when the tumors reached a maximum volume of 1600 mm^3^.

### Measurements and statistical analysis

Statistical analyses were conducted using SAS^®^ v9.4 software. Categorical variables were described as percentages per class and continuous variables as means and standard deviations. Overall survival (OS) and tumor growth (TG) were the endpoints. OS was defined as the time from graft implantation until the tumor reached or exceeded 1000 mm^3^, or until a tumor ulceration occurred, or until a serious deterioration of clinical status occurred. If the mouse did not meet one of these conditions at the end of the experiment, it was considered as surviving for the statistical analysis, although euthanized. OS was analyzed using Kaplan-Meier curves and *log-rank* tests. Tumor volumes were calculated using the following formula: (L × W²)/2, where L is the length and W is the width. TG was analyzed using mixed models for repeated measurements (MMRM), with mice as a random effect, group, time and the interaction between group and time as fixed factors. Correlation structures [compound symmetry (CS) and unstructured (UN)] were tested, and the best structure was considered for the model.

Two-sided *p*-value ≤ 0.05 were retained on the day of treatment/group interaction for statistical significance, before considering any daily difference between groups. A *p*-value ≤ 0.1 was considered as a trend marker. In figures the significances are thus represented: no stars: no statistical difference (*p*-value > 0.05); one star (*): 0.05 ≥ *p*-value > 0.01; two stars (**): 0.01 ≥ *p*-value > 0.001; three stars (***): 0.001 ≥ *p*-value > 0.0001; four stars (****): 0.0001 ≥ *p*-value.

### Immunophenotyping

Five mice per group were randomly euthanised, the tumors sampled and prepared for immunophenotyping. Briefly, tumors were disrupted using the GentleMACS™ (Miltenyi Biotech) and the corresponding mouse tumor preparation kit (130-096-730, Miltenyl Biotec), before labelling to detect cell subsets with markers such as for T effectors cells (CD45+CD3+CD4-CD8+), or M1 TAM cells (CD45+CD3-CD11b+CD68+CD206-); Antibodies used are listed **Table 1**. The labelled cells were processed using a LSRII™ (BD) and the populations analyzed by FlowJo™.

**Table 1:** References of antibodies used for the immunophenotyping.

Reagent	Source	Identifier	PRID
CD45	BD	564279	AB_2651134
CD3	Thermo	58-0032-82	AB_11217479
CD4	BD	563106	AB_2687550
CD8	BD	750024	AB_2874242
CD11b	Thermo	48-0112-82	AB_1582236
CD68	Thermo	14-0688-82	AB_11151139
CD206	Biolegend	141732	AB_2565932

### Proteins extraction for proteomic analysis

CT26, CMT-93, LTPA, CT26-SH, CMT-93-SH, and LTPA-SH cells were washed and lysed, and the proteins were precipitated with an organic solvent. The protein pellet was denatured in 8 M urea, reduced with 20 mM dithiothreitol (DTT) for 40 min at 56°C and alkylated with 50 mM iodoacetamide (IAA) for 40 min in the dark at room temperature. Overnight digestion was performed at 37°C using trypsin at a ratio of 1/20 (enzyme/total protein; w/w).

AQTBEADS (Anaquant, Lyon, FR) used for the standard-based calibration curve, was added to each sample after digestion. The samples were acidified with formic acid (0.1% w/v final) and desalted on HLB columns (Waters, Manchester, UK) according to the manufacturer’s protocol prior to mass spectrometry (MS) analysis.

### Mass spectrometry analyses

Large-scale analysis in data-dependent analysis (DDA) mode was performed on a Q-Exactive HF instrument (Thermo-Fisher Scientific, San Jose, CA) coupled to an RSLC Ultimate 3000 nano system liquid chromatography system (Thermo-Fisher Scientific, San Jose, CA). The MS instrument was operated in positive mode with the following parameters: ionization voltage +1.8 kV, temperature of the ion transfer capillary 250°C, and S-lens level 60 arbitrary units. For MS acquisition, the resolution was set at 60 000, AGC target at 3.106 maximum IT at 60 ms, and scan range at 375-1600 m/z. For MS2 acquisition, the instrument was operated at a resolution 15 000, with an AGC target of 1.105 and a maximum IT of 60 ms. The TOP20 parent ions were fragmented, with an isolation window of 2 m/z, NCE set up at 27, and dynamic exclusion at 10 s. For peptide separation, a PepMapTM RSLC C18 analytical column, 2 µm, 0.075 mm ID × 500 mm (Thermo-Fisher Scientific, San Jose, CA) was used. Solvent A was water containing 0.1% formic acid and solvent B was ACN containing 0.1% formic acid. The peptides were eluted with a gradient of 3 to 40% solvent B over 60 min at a flow rate of 300 nL/min. 250ng sample was loaded onto the column (calibration curve from 1 to 500 fmol injected).

### Data processing

A *mus musculus* protein sequence database containing the reviewed sequences was built to retrieve proteins expressed in murine cells. MS/MS spectra were assigned to peptide sequences using a database search strategy with the X!Tandem search engine. Trypsin was used as the enzyme and two missed cleavages were allowed. Cysteine modification was set as a fixed modification, whereas mono-oxidation of methionine was set as a variable modification. The mass tolerances of the precursors and fragment ions were set to 10 and 20 ppm, respectively. A decoy strategy was used to ensure a false discovery rate (FDR) of < 1%. The validation step was performed using Proline software v1.6.1 (http://www.profiproteomics.fr/proline/). For filtering at the peptide spectral match (PSM) level, the score threshold was set at 20 for rank 2 peptides. For filtering at the protein level, proteins with at least two identified peptides were used, and one specific peptide was retained. The quantification step (APEX intensity) was performed using Proline software, with default parameters recommended by the developers. No normalization was selected in the Proline software parameters.

(http://www.profiproteomics.fr/wpcontent/uploads/2019/03/ProlineSuite_UserGuide_2.0.docx.pdf). TOP3 results extracted from Proline were processed using the HCPprofiler application (Anaquant, Lyon, FR) to extract the injected protein quantity based on the AQTBeads calibration curve.

## Results

### One cell line based STC vaccine inhibit tumor growth and improve survival

**Figure 3:**
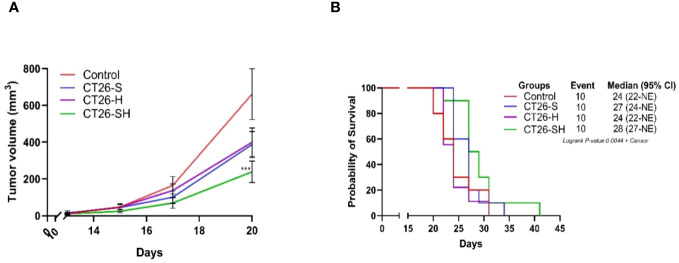
Effect of stimulated and/or haptenated CT26 vaccine. **(A)** Tumor growth curves of tumor-bearing mice (n = 10 per group). Administration of the CT26-SH (G4) vaccine significantly retarded tumor growth compared to that in the control group (G1) (p-value=0.003, Student t test at day 20). The CT26-SH group showed a better effect than the other two treatment groups (G2: CT26-S and G3: CT26-H) on day 20. ***: 0.001 ≥ p-value > 0.0001. **(B)** Kaplan-Meier survival analysis of tumor-bearing mice (n = 10 per group). With the best survival time for CT26-SH group (G4) compared to the control group (G1) (p-value=0.0748, log-rank test), CT26-S vaccinated group (G2), and CT26-H vaccinated group (G3). Log-rank test comparing all groups with each other, p-value =0.0044. NE: not evaluable; the bounds of confidence indices (CI) cannot be calculated. At Risk table shown in **Supplementary Figure 1A**.

To investigate the relevance of the STC platform to produce therapeutic vaccine, we tested in a first study (A), the efficacy of 1CL (CT26)-based STC vaccine in syngeneic models generated by subcutaneously injecting of CT26 cells into the mice. Tumor growth (TG) levels were monitored for up to 41 days. As shown in **Figure 3A**, CT26-S vaccine reduced TG levels with an average tumor volume of 386 mm^3^ on day 20 (elimination of the first animal from the control group for ethical recommendation) compared to 661 mm^3^ in the control group. In addition, treatment with CT26-H vaccine showed a growth inhibitory effect similar to that of CT26-S vaccine. Administration of the CT26-SH vaccine had the strongest tumor-reducing effect, with an average tumor volume of 238 mm^3^ on day 20 (*p*-value=0.003 vs Control group, Student t test). Consistently, survival time was longer for mice vaccinated with CT26-SH than mice vaccinated with CT26-H or CT26-S (CT26-SH mOS 28 days vs CT26-H mOS 24 days, *p*-value=0.0219, *log-rank* test) (**Figure 3B**). These results showed that the combination of stress and haptenation of CT26 cell line promoted better effects than the single stimulation or haptenation.

### Immunostimulant effect

**Figure 4:**
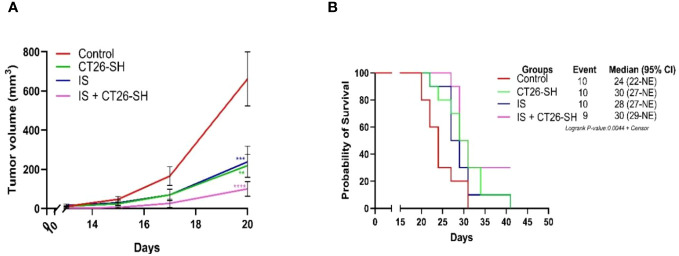
Immunostimulant associated to CT26-SH vaccine. **(A)** Tumor growth curves of the tumor-bearing mice (n = 10 in the control group: G1, n = 10 in the CT26-SH group: G4, n = 10 in IS group: G5 and n = 9 in the IS + CT26-SH group: G6). Administration of the CT26-SH vaccine + IS (G6) significantly retarded tumor growth compared to control group (G1) (p<0.0003, Student’s t-test at day 20). ns: no significant. **: 0.01 ≥ p-value > 0.001; ***: 0.001 ≥ p-value > 0.0001; ****: 0.0001 ≥ p-value. **(B)** Kaplan-Meier survival analysis of tumor-bearing mice (n = 10 in the control group: G1, n = 10 in the CT26-SH group: G4, n = 10 in IS group: G5 and n = 9 in the IS + CT26-SH group: G6). Survival analyses indicated the best survival time in the IS + CT26-SH group compared to the control group (p-value=0.0046, log-rank test), IS group (p-value=0.0220, log-rank test) and CT26-SH vaccinated group (p-value=0.0748, log-rank test). Log-rank test comparing all the groups with each other, which shows the significance of one of the groups on this parameter log-rank test p-value=0,0044. NE, not evaluable, the bounds of confidence indices (CI) cannot be calculated. IS, immunostimulant: 15 mg/kg CP and 5 µg mGM-CSF. At Risk table shown in **Supplementary Figure 1B**.

To enhance the antitumor effect of the STC vaccine, we combined CT26-SH vaccine with immunostimulants (IS). Mice developing CT26 tumors were treated with the CT26-SH vaccine and IS: 15 mg/kg cyclophosphamide (CP) and 5 µg mGM-CSF, as described in Materials and Methods. CT26-SH vaccine + IS significantly reduced TG and induce overall survival compared to animals treated with CT26-SH vaccine alone (100 mm^3^ vs. 238 mm^3^ respectively, p<0.0003, Student’s t-test at day 20) (**Figure 4**). IS + CT26-S vaccine and IS + CT26-H vaccine were also tested, and no significant results were reported (**Supplementary Figure 2**). Adding another IS, such as BCG, did not provide any additional effect when used in combination with CP and mGM-CSF as adjuvant for the CT26-SH vaccine (**Supplementary Figure 2**).

### Three cell lines-STC based vaccine efficacy

The aim of the second set of experiments (Study B) was to test the efficacity of STC vaccine expressing a wider repertoire of tumor-related antigen. We thus developed a novel vaccine based on the stimulation of three different cell lines: two CRC murine cell lines (CT26 and CMT-93) and one pancreatic adenocarcinoma murine cell line (LTPA). In Study B, CT26 cells were injected into mice as in Study A, and TG was monitored for up to 51 days for the different groups.

**Figure 5:**
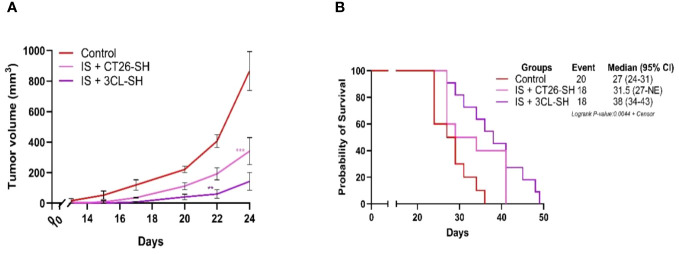
Effect of three cell lines-based vaccine, stimulated and haptenated. **(A)** Tumor growth curves of tumor-bearing mice (n = 20 in the control group: G’1, n = 18 in the IS + CT26-SH group: G’2 and n = 18 in the IS + 3CL-SH group: G’3). Administration of the IS + 3CL-SH vaccine significantly retarded tumor growth compared to that in the control group (p-value =0.0067, Student’s t-test at day 22). **: 0.01 ≥ p-value > 0.001; ***: 0.001 ≥ p-value > 0.0001. **(B)** Kaplan-Meier analysis of the survival of tumor-bearing mice (n = 20 in the control group, n = 18 in the IS + CT26-SH group and n = 18 in the IS + 3CL-SH group). Survival analyses indicated that the IS + 3CL-SH group had the best survival time compared to the control group (p<0,0001, log-rank test) and the IS + CT26-SH vaccinated group (p-value=0,0023, log-rank test). Log-rank test comparing all the groups with each other, which shows the significance of one of the groups on this parameter log-rank test p-value < 0,0001. NE, not evaluable, the bounds of confidence indices (CI) cannot be calculated. 3CL-SH: three cell lines-based vaccine, stimulated and haptenated. IS, immunostimulant: 15 mg/kg CP and 5 µg mGM-CSF. At Risk table shown in **Supplementary Figure 1C**.

Vaccination was systematically combined with IS (15 mg/kg CP and 5 µg mGM-CSF). Data from Study B showed that the 3CL-based vaccine had a largest effect compared to 1CL-based vaccine on TG and overall survival (OS) (**Figure 5**). As shown in **Figure 5A**, IS + 3CL-SH vaccine reduced TG with an average tumor volume of 150 mm^3^ on day 24 (elimination of the first animal from the control group for ethical recommendation) compared to 341 mm^3^ for the group treated with IS + CT26-SH vaccine (*p*-value=0.0002, Student’s t-test). The mOS was significantly longer in mice treated with IS + 3CL-SH vaccine (38 days) than in those treated with IS + CT26-SH vaccine (31.5 days) (*p*-value=0.05, *log-rank* test) (**Figure 5B**). In additional group, mice received 3CL-SH vaccine more frequently (2 times per week for 4 weeks on D7, D10, D14, D17, D21, D24, D28, and D31) in combination with IS. No significant efficacy was observed for TG and OS compared to the group treated by IS + 3CL-SH vaccine one injection per week at D7, D14, and D21.

### Antigen expression on the three cell lines-based vaccine

**Figure 6:**
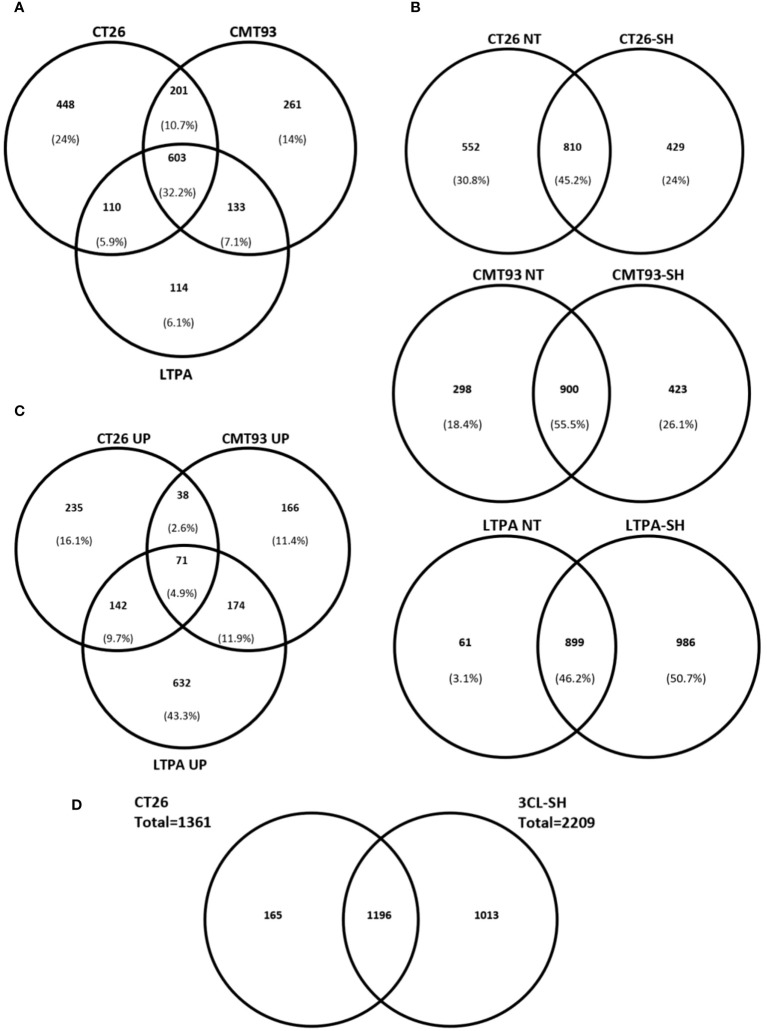
STC vaccine at proteomic level. **(A)** Venn diagram comparing the identified proteins in each cell models for untreated samples. **(B)** Venn diagram showing percentage of specific proteins of each cell line before and after simulation and haptenation. **(C)** Up-regulated proteins after simulation and haptenation, Venn diagram showing the percentage of specific and shared proteins for each cell line. **(D)** Venn diagram showing the overlapped proteins between CT26 cell line and the 3CL-SH vaccine. UP, up-regulated; NT, untreated.

We established a proteomic approach of various drug products (vaccine versions). Data of protein expression, from LC/MS-MS of CT26, CMT-93, and LTPA-untreated cells showed that 32% of the identified proteins were common in all cell lines. In contrast, 44% of the total identified proteins were specific for each cell line (24% for CT26, 14% for CMT-93, and 6,1% for LTPA of total expressed proteins) (**Figure 6A**). In addition, proteins were specifically expressed in each cell line after simulation. CT26-SH specifically expressed 429 proteins and shared 810 proteins with the untreated CT26. Similarly to CT26, CMT-93-SH specifically expressing 423 proteins and share 900 proteins with untreated CMT-93 cells. LTPA-SH had 899 and 986 proteins shared with untreated cells or expressed specifically after simulation, respectively (**Figure 6B**).

Among these proteins, some were up-regulated after simulation of the 3 cell lines: CT26, CMT-93, and LTPA. Overexpressed proteins were specific for each cell line (16.1% for CT26, 11.4% for CMT-93, and 43.3% for LTPA of the total overexpressed proteins) (**Figure 6C**). Several tumor-related proteins overexpressed in one or more cell lines are summarized in **Table 2**, as FAS-mediated apoptosis may play a role in the induction of peripheral tolerance in antigen-stimulated suicide of mature T-cells. Interestingly, over 50% of the LTPA-SH cell line overexpressed major proteins of interest, such as Lysosomal Associated Membrane Protein 1 (Lamp 1) and multidrug resistance proteins A and B (MDR 1A and B), which efflux drugs across the membrane and are implicated in multidrug resistance mechanisms. Globally, we observed that thermal stress and haptenation resulted in the overexpression of 30 to 68% of the proteins in these cell lines. Overall, 1196 proteins expressed by the CT26 cell line were shared with the 3CL-SH vaccine (**Figure 6D**).

**Table 2:** Tumor-related proteins, up-regulated in one or more cell line after stimulation and haptenation.

	-SH / NT fold change				
Description	CT26	CMT93	LTPA	Link to	Ref
**ENOA**	**13**			**Identified as an autoantigen. Mediates extracellular matrix degradation**	**PMID: 27814656**
**NUP54**	**241**			**Play an important role in the assembly and functioning of the nuclear pore complex. Regulates the movement of macromolecules across the nuclear envelope**	**PMID: 31164343**
**NUP85**		**114**			
**NU214**			**218**		
**CDC37**	**241**		**36**	**Cell division cycle control protein. Play a critical role in directing Hsp90 to its target kinases.**	**PMID: 8666233**
**HSPB1**		**4**	**109**	**Correlated with poor clinical outcome in multiple cancers. Promote cancer cell proliferation, metastasis and protect cancer cells from apoptosis.**	**PMID: 17661394**
**LAMP2**			**327**	**Required for efficient MHCII-mediated presentation of exogenous antigens**	**PMID: 20518820**
**LAMP1**			**182**		
**CDK1**	**241**	**229**	**473**	**Member of the Ser/Thr protein kinase family. Plays a key role in the control of the eukaryotic cell cycle**	**PMID: 17700700**
**CDK4**			**182**	**Member of the Ser/Thr protein kinase family. Mutations in this gene were found to be associated with tumorigenesis of a variety of cancers.**	**PMID: 26977878**
**CDK13**			**1164**	**Member of the Ser/Thr protein kinase family. Play a role in mRNA processing and may be involved in regulation of hematopoiesis.**	**PMID: 17261272**
**TTK**			**36**	**Predictor of poor prognosis in colorectal cancer. Mediate multiple drug resistance in cancer**	**PMID: 33754407; PMID: 27072896**
**EPHA2**		**689**	**582**	**Class of receptor tyrosine kinases. Contribute to modulatory processes controlling carcinogenesis and tumor progression. expression in highly proliferating epithelial cells**	**PMID: 15054110**
**UBE2N**	**120**		**72**	**Play a role in the control of progress through the cell cycle and differentiation. Contributes to the survival of cells after DNA damage.**	**PMID: 22424771**
**UBE2C**		**114**		**Essential factor of the cell cycle-regulated ubiquitin ligase that controls progression through mitosis.**	**PMID 34620747; PMID 34950739**
**MTA2**		**2**		**Central component of the nucleosome remodeling and histone deacetylation complex. Regulate cytoskeletal and motility pathways**	**PMID: 33754407**
**MTA1**			**291**	**Plays an important role in tumorigenesis, tumor invasion, and metastasis.**	**PMID: 19837670; PMID: 20682799**
**BIRC6**			**218**	**Anti-apoptotic protein which can regulate cell death by controlling caspases**	**PMID: 15485903**
**PCNA**	**603**			**Involved in the control of eukaryotic DNA replication. Play a key role in DNA damage response**	**PMID: 19443450**
**ABCB7**			**36**	**Regulate apoptotic and non-apoptotic cell death by modulating mitochondrial ROS**	**PMID: 31511561**
**CPSF3**			**109**	**Upregulated in CRC tissues. Correlated with unfavorable prognosis**	**PMID: 22237626**
**TNPO1**	**3**			**Target nuclear proteins to the nucleus. Upregulated in cancer. Potential tumor biomarker**	**PMID: 22237626; PMID: 36175880**
**TNPO3**			**72**	**Nuclear import receptor. Expression correlated with cancer. Could serve as a potential diagnostic biomarker**	**PMID: 34703650**
**FAS**	**724**		**582**	**A transmembrane protein which can induce cell death by apoptosis**	**PMID: 34675185**
**MDR1A**			**291**	**Efflux drugs across the membrane and implicated on multidrug resistant cells**	**PMID: 1969610**
**MDR1B**			**254**		

NT, untreated.

The highlighted boxes are for the possibility of making comparisons beyond the two conditions, SH and NT.

### STC vaccine efficacy in PD-1-resistant model

**Figure 7:**
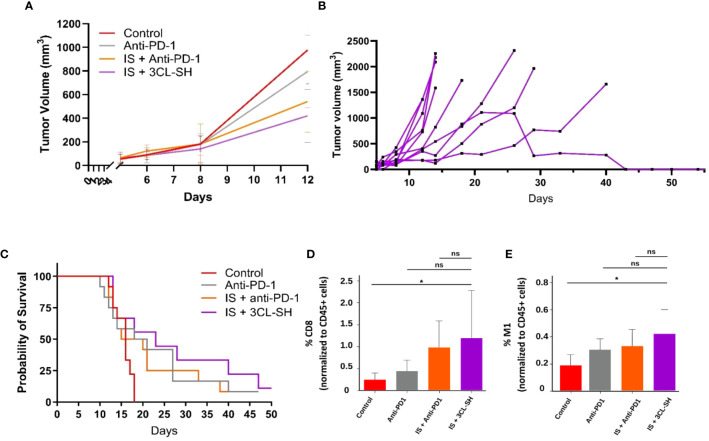
Effect of the 3 cell lines-STC based vaccine on MC38 PD1-R. **(A)** Tumor growth curves of tumor-bearing mice (n = 15 per group, G’’1: control group, G’’2: anti-PD-1 group, G’’3: IS + anti-PD1 group and G’’4: IS + 3 CL-SH group. No statistical difference between the groups was observed at D12. **(B)** Individual curves for the tumor growth of mice of the G’’4: IS + 3 CL-SH group. **(C)** Kaplan-Meier analysis of the survival of tumor-bearing mice (n = 15 per group G’’1: control group, G’’2: anti-PD-1 group, G’’3: IS + anti-PD1 group and G’’4: IS + 3 CL-SH group). Significant improvement of the survival of mice bearing MC38- PD-1R tumor treated by 3 CL-SH+IS vaccine vs control (Log-rank Mantel-Cox test, p-value=0,0368). **(D)** CD8+ infiltration in the TME (Tumor Micro-environment) at D14 (n=5/group). Group treated with IS + 3 CL-SH vaccine displays a significant increase in the infiltration of cytotoxic CD8+ T cells when compared with the control group (p-value =0.045, Student’s t-test). ns, non-significant. **(E)** TAM 1 infiltration in the TME (Tumor Micro-environment) at D14 (n=5/group) showing increase in anti-tumoral M1 macrophages in the group treated with IS + 3 CL-SH vaccine vs control (p-value =0.02, Student’s t-test). 3CL-SH, three cell lines-based vaccine, stimulated and haptenated. ns, non-significant. IS, immunostimulant: 15 mg/kg CP and 5 µg mGM-CSF. *: 0.05 ≥ p-value.

A MC38 anti PD-1-Resistant model has been developed (10) and vaccine doses from study B were used. As shown in **Figure 7A**, 3CL-SH vaccine reduces tumor size compared to control group at D12 (date of the euthanasia of the first mice reaching the ethical volume of 1 600mm^3^). No statistical difference between the groups was observed at D12. The observation of the individual responses within group 3CL-SH (**Figure 7B**) shows a complete regression of the tumor volume for one mouse which had reached more than 1 000 mm^3^ tumor volume. As observed in studies A and B, immunostimulant (mGM-CSF and CP) enhance the anti-tumor effect of the vaccine (G’’3). The group treated with IS + 3CL-SH significantly improves the survival of mice bearing MC38 anti-PD-1-Resistant tumor model comparatively to the control group (*p-value*=0.00368, Log-rank Mantel-Cox test) (**Figure 7C**).

The immunophenotyping data (**Figures 7D, E**) show significant increase in the infiltration of anti-tumor populations: CD8+ T cells (*p*-value =0.045, Student’s t-test) and M1 macrophages (*p*-value =0.02, Student’s t-test) in mice treated with IS + 3CL-SH vaccine vs Control group.

### STC vaccine safety data

In these three studies, all the mice were observed daily to detect any toxic effect of the product or necrosis of the tumors. The weights and behavior of the mice were monitored throughout the study. The injection sites were monitored for inflammatory reactions, and tumor size (length: L; width: W) was measured using a caliper three times a week. The mice were euthanized at the end of the study or, before the tumor reached or exceeded 1000 mm^3^ (for study A and B) or 1 600 mm^3^ (for study C), if tumor ulceration occurred, or in case of serious deterioration of clinical status. Antitumoral activity was assessed by measuring tumor volume and median survival. For each of these 3 studies, no side effect or inflammatory reaction towards the 3CL-SH is evidenced. Overall, seven mice were excluded from the statistical analysis for different reasons, all unrelated to direct toxicity: unsuccessful grafting, ulceration before reaching the tumor size limit, and unexpected death at D22 for study B (Supplementary safety data).

## Discussion

Although treatment efficacy has improved over the past decade, the survival rate of patients with CRC remains low. Recent tremendous progress has established immunotherapy as a treatment option and has opened multiple ways to address therapeutic challenges in CRC, but they are still limited concerning the panel of antigen and have manufacturing challenges that may be a barrier for the patient’s access (12). Accordingly, we aimed to develop a platform overcoming these challenges and easily produce therapeutic cancer vaccine based on the stimulated tumor cells (STC) technology. This concept relies on proprietary cell-based products stimulated *in vitro* to induce cancer-related antigen expression and improve their immunogenicity. We defined stress applied in the production as thermal stress and irradiation. Thermal stress proteins such as HSP have been used in vaccine strategies. However, HSP-isolated vaccines have not been confirmed in clinical studies (28). Thus, STC vaccines overexpressing thermal stress protein would be an interesting way to overcome this challenge. In addition, radiotherapy is known to induce an abscopal effect, a systemic response mediated by the immune system to local irradiation, comparable to an “*in situ* vaccine” (29). Here, irradiation of the vaccine was assumed to generate an abscopal-like effect, with immune priming supported by the exogenous irradiated vaccine. This triggers an immune reaction against tumor cells that express the same tumor antigens. We assessed the expression of cancer-related antigens through transcriptomics and proteomic analysis. Subsequently, we compared the proteome of the STC drug product with *in vivo* tumor biopsies database to validate the significance of the expressed targets. Haptenation step is also added to the production strategies. Haptenated-vaccine efficacy have been already tested in melanoma patients (25) and recurrent glioblastoma (Gliovac, ERC Belgium) where clinical data on this indication shows a mOS of 7.8 months and mPFS of 2.4 months. Gliovac in is current study on real life patients (compassionate use) presented a first series of patients with a mOS of 19,6 months (95% CI: 8.42- NR) and the mPFS of 9.14 months (95% CI: 6.15-20.19). The 10 most advanced patients have an mOS of 30.64 months. Based on these impressive results, the FDA granted a Fast track to start Phase III study (30, 31).

In this study, we determined production strategies to produce STC vaccine. We started with single stress or simple haptenation. Then stressed and haptenated vaccine was first produced using only one cell line or three cell lines and tested in different CRC murine models. The murine CT26 CRC model mimicking pMMR CRC and evaluating sensitization strategies based on immunogenic chemotherapy (5-FU/oxaliplatin, trifluridine/tipiracile/oxaliplatin) (32, 33) or radiotherapy has been shown (34). These strategies are currently being clinically evaluated in pMMR patients with CRC and have shown encouraging results (35). In Study A, the results strongly endorse the effectiveness of STC technology as a promising platform for producing therapeutic vaccines. Subsequently, we evaluated the efficacy of a 3CL-based vaccine to provide better coverage of the heterogeneity of tumor cells and the potential tumor antigens identified in digestive system cancers. We employed pancreatic and colorectal cancer cell lines, which have demonstrated shared neoantigens and tumor-associated antigens with CRC cell lines. (36). According to this study, the 3CL-based vaccine with the largest panel of tumor-related antigens is more effective in reducing tumor growth than the 1CL approach. This suggests that the immune system is better activated by the 3CL-based vaccine. The broader range of antigens presented by 3CL is likely responsible for this effect, as it is more representative of the cellular diversity encountered in mouse tumors. This observation was confirmed by a proteomic approach, which showed that the 3CL-based vaccine had the highest number of proteins identified compared to the 1CL-based vaccine. Indeed, proteomic analysis showed overexpression of tumor-related proteins such as TTK, MTA or MDR1. Peptides and mRNAs of these proteins have been tested in several studies as potential targeted T cell therapies or cancer vaccines (**Table 2**) (37, 38).

Contrary to CAR T cell therapy, peptide, and mRNA vaccines that can offer up to eight neoantigens (39), STC technology offers a pool of antigens with strong immunogenicity, ensuring better education for the patient’s immune system, which aims to prime T-lymphocytes to recognize and eliminate tumor cells. In contrast to ‘personalized therapies’, allogenic vaccines can be used to treat many patients by covering several tumor antigens (40), plus an easier production process. The STC vaccine technology can be transposed, and the same principles can be applied to develop several human vaccines targeting different types of solid tumors. However, the selection of cell lines composing the vaccine is a significant challenge to have a vaccine that is match the extensive diversity and heterogeneity of tumor patients for a selected indication. Tumors in patients exhibit extensive heterogeneity, capturing this diversity in a limited set of cell lines is difficult, leading to a vaccine that is effective against only a subset of tumor types. This process could be improved to accelerate drug discovery development and highlights the importance of developing strategies such as stimulation and haptenation to encompass tumor heterogeneity.

In addition, we evaluated the 3CL-based vaccine in allogenic MC38 anti-PD-1-Resistant model. Mice that received the vaccine presented 60% of responders including a complete response and provided a gain of survival and an increase in anti-tumoral TME immune population (CD8+ and M1 macrophages). This MC38 anti-PD-1-R resistant model usually displays a strong decrease of CD8+ T cells infiltration and a high level of TAM M2 like cells (10). The construction of this model induces a selected pressure with the anti-PD-1 increasing dramatically PMN-MSDC (10) and plays a role in the induction of secondary resistance can explain the absence of tumor volume regression by negative action of the TAM M2 with anti-PD-1. Immunophenotyping assays were limited in these studies. Additional *in vivo* proof-of-concept experiments should be conducted to gain a better understanding of the vaccine’s mechanism of action. Overall, the 3CL-SH vaccine was tested in both MSS (CT26) and MSI (MC38) models. Since MSS CRCs constitute the majority of human CRC cases and are typically less responsive to immune checkpoint inhibitors, the CT26 model is crucial for developing new immunotherapy strategies. Tumor resistance is a significant concern in oncology, making the development of cancer vaccines to prevent resistance a critical goal in immunotherapy. Additionally, the MC38 resistance model is important for testing the allogeneic potential of the 3CL-SH vaccine and to test its potential in acquired immunotherapy resistance model that can be observed in relapse patients following immune check point treatment. Utilizing both MSI and MSS models in cancer vaccination strategies is essential for creating effective treatments across diverse genetic backgrounds and immune environments, ultimately leading to more successful cancer therapies. Although the results in mouse models are promising, predicting efficacy in patients requires exploration in different models due to the use of a murine surrogate in syngeneic models. Human vaccines based on STC technology should be tested in alternative models, such as ex vivo immune response activation tests, to better assess biological and clinical responses.

The association of the STC vaccines with immunostimulant, boost dendritic cells (DCs) uptake and naïve T-cells activation via multi-specific tumor antigens (41). mGM-CSF and CP reinforced the vaccine’s effect. They were selected because CP can directly induce tumor cell death and restore antitumor immunity by decreasing the number and alleviating the suppressive function of regulatory T-cells (31, 42); whereas GM-CSF promotes survival, growth and differentiation of various immune cells as well as recruitment of dendritic cells at the site of vaccine injection (43). Both are recognized as potent adjuvants for immunotherapy and their therapeutic efficacy has been demonstrated in numerous preclinical studies (44). However, CP and GM-CSF can also have a deleterious impact on anti-tumor immunity if the doses and administration schedule are not optimized, which can partially explain the heterogeneous results of different clinical trials (45). Compared to GVAX strategy where vaccine cells secrete GM-CSF with the difficulty to control the level of *in vivo* secretion, our strategy, using fixed low dose of GM-CSF, allows us to control the dose of cytokines delivered to the patient (36, 46). Although the combination of whole-cell vaccines and BCG was tested for ONCOVAX, it did not provide any further benefits for STC vaccine (13). BCG immunotherapeutic potential is supposed to rely on the activation and exhaustion of tumor-specific T-cells, however, the exact mechanism of action remains unclear (47). Manrique-Rincon et al. recently showed that the antitumor effects of GVAX were potentiated by inhibiting the immunosuppressive phenotype of regulatory T cells using a small antisense RNA targeting the Foxp3 gene (48). Therefore, combination of STC vaccine with different IS, checkpoint inhibitors and chemotherapy should be investigated since a strong biological rational to combine exist. Further improvements could be made to the STC manufacturing platform, particularly during the adherent cell amplification phases, to increase batch size. Additionally, the risk of environmental contamination should be mitigated by expanding the use of closed systems to ensure aseptic process conditions.

The combination of STC vaccine with IS in this regimen is safe with no significant safety concerns were observed. In addition of these studies, a toxicity study conducted in parallel on 76 mice, with repeated SC administration of vaccine and mGM-CSF associated with intraperitoneal CP for 8 weeks, showed no treatment-related deaths, no test item-related clinical signs, no local reactions, and no effects on body weight or food consumption.

## Conclusion

STC vaccine based on stimulations and haptenation of CRC tumor cells and guided by proteomic demonstrated a significant anticancer effect in mice treated with immunostimulant and confirmed the superior efficacy of the three cell lines STC vaccine compared to a single cell line STC vaccine. These proof-of-concept studies confirmed the potential of the STC approach to treat CRC cancer pMMR/MSS or resistant to anti-PD-1 by educating and activating efficiently the immune system. Moreover, compared with other immunotherapies of identical principles, the manufacturing processes for STC vaccines are more time-saving and cost-effective. The technological platform of STC vaccine has the potential to be used in the clinical setting for the treatment of patients with CRC.

## Data availability statement

The original contributions presented in the study are included in the article/**Supplementary Material**. Further inquiries can be directed to the corresponding author.

## Ethics statement

Ethical approval was not required for the studies on humans in accordance with the local legislation and institutional requirements because only commercially available established cell lines were used. The animal study was approved by Ethics Committee for Animals (UCBL1, Lyon, France, approval #DR2015-29) the Animal Ethics Committee CECCAP of Lyon. The study was conducted in accordance with the local legislation and institutional requirements.

## Author contributions

GA: Formal analysis, Investigation, Methodology, Supervision, Validation, Visualization, Writing – original draft, Writing – review & editing. CT: Formal analysis, Investigation, Supervision, Validation, Writing – original draft, Writing – review & editing, Conceptualization, Methodology. JT: Investigation, Methodology, Writing – review & editing. FD: Writing – review & editing, Investigation. BB: Formal analysis, Writing – review & editing, Software. CB: Investigation, Writing – review & editing. EL: Visualization, Writing – review & editing. MB: Writing – review & editing, Methodology. LC: Investigation, Supervision, Validation, Writing – review & editing, Conceptualization, Methodology, Visualization. BP: Funding acquisition, Investigation, Methodology, Supervision, Validation, Visualization, Writing – original draft, Resources. PB: Funding acquisition, Methodology, Supervision, Visualization, Writing – review & editing, Formal analysis, Investigation, Project administration, Resources, Validation. CG: Investigation, Supervision, Visualization, Writing – review & editing. LA: Visualization, Writing – review & editing, Validation. FG: Supervision, Writing – review & editing, Visualization.
