# Supplementary material for: Efficacy of novel allogeneic cancer cells vaccine to treat colorectal cancer
Source: Front Oncol. 2024 Jul 24;14:1427428. doi: 10.3389/fonc.2024.1427428 (PMC11303197; doi:10.3389/fonc.2024.1427428)
Supplement: Supplementary file 1 [file DataSheet_1.pdf]

Supplementary file

Supplementary figure and table

Supplementary table 1:

Cells	Samples	MFI	Corrected MFI by Isotype	Ratio Cells-SH/Cells-WT
CMT93-SH	Iso IgG1	284	14067	5,00
	hsp70	14351		
CMT93	Iso IgG1	95	2814	
	hsp70	2909		
CT26-SH	Iso IgG1	301	13589	4,35
	hsp70	13890		
CT26	Iso IgG1	153	3125	
	hsp70	3278		
LTPA-SH	Iso IgG1	323	17694	4,34
	hsp70	18017		
LTPA	Iso IgG1	135	4073	
	hsp70	4208		

HSP70 expression after thermal stress + irradiation 10Gy and haptenization. Samples were analyzed by flow cytometry. Shown is the mean of the median fluorescence intensity (MFI) (isotype subtracted) relative to unstimulated.

Supplementary Figure 1

A

Days	1	22	24	27	31	41
Control	10	8	6	3	2	0
CT26-S	10	10	10	6	2	0
CT26-H	10	10	5	2	1	0
CT26-SH	10	10	10	9	3	1

B

Days	1	22	24	27	31	41
Control	10	8	6	3	2	0
CT26-SH	10	10	10	9	3	1
IS	10	10	10	9	3	1
IS + CT26-SH	9	9	9	9	5	3

C

Days	1	27	29	31	34	36	41	45	48	49
Control	20	12	10	6	4	2	0	0	0	0
IS + CT26-SH	18	18	9	6	7	7	7	0	0	0
IS + 3CL-SH	18	18	16	14	12	11	10	7	5	2

Supplementary Figure 2

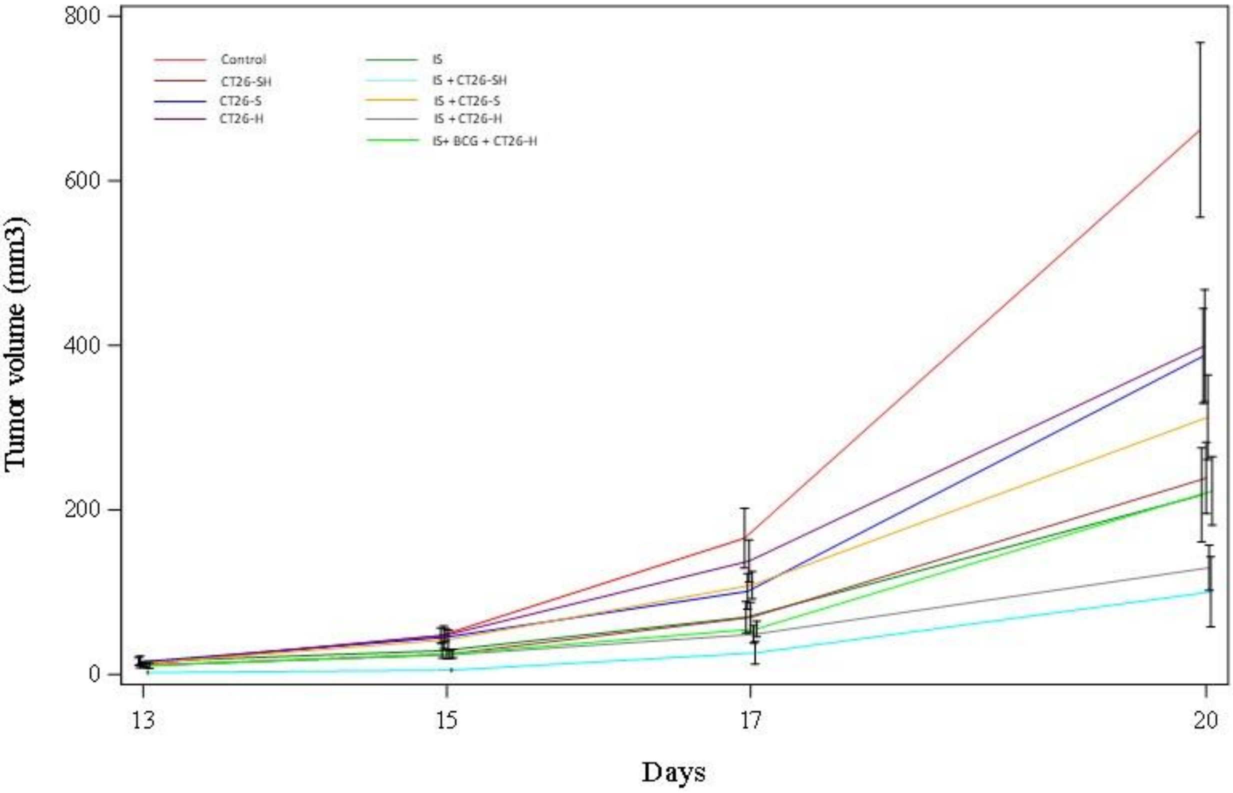

Effect of physically stimulated and / or haptenized CT26 vaccine + / - immunostimulant. Tumor growth curves.

Supplementary materials and methods:

Safety:

Overall, 7 mice were excluded from studies for different reasons. One mouse for unsuccess of grafting G’4 (IS + 3CL-SH x2), one mouse for ulceration before reaching the limit tumor size on G’3 (IS + 3CL-SH) and 5 mice have unexpected death at D22 in groups: G’2 (IS + CT-26-SH) and G’4 (IS + 3CL-SH x2). About these premature deaths, there was no sign allowing the prediction of this event the day before and after the injections. The examination of the cadavers showed no element explaining these deaths. The environmental conditions, food, water, temperature, and hygrometry were normal. To evaluate if these deaths could be attributed to an immune reaction, body temperature before and after injection and aspect of injection zone were monitored. The body temperature variations were non-significant during the 3 previous injections and no signs indicating an adverse reaction to the vaccine have been observed at the level of vaccine injection area. It is impossible to conclude that the vaccines or the immunostimulants causes these deaths because they occurred in different groups; but it is important to note that there were no deaths in 2 groups received vaccine and immunostimulant (G’1: Control/vehicle and G’3 (IS + 3CL-SH)).

Flow cytometry:

After at least 24 hours at -80°C, cells (CMT93; CMT93-SH; CT26; CT26-SH; LTPA and LTPA-SH) were thawed at 37°C then fixed with 4% paraformaldehyde for 20 minutes at 4°C. Cells were resuspended in PBS 1% BSA at a density of 2.10^6 cells/ml. 100 µl of each cell’s suspension were used for staining using 2µl anti-cmHSP70.1 (Klinikum, TU München) or 2µl IgG1 isotype (santa Cruz). 100 µl PBS 1% BSA were added on each sample after incubation at 4°C for 30 minutes. Cells were then washed by PBS. All samples were analyzed using Attune (Thermo) flow cytometer.
